# Interplay between the Adaptive Immune System and Insulin Resistance in Weight Loss Induced by Bariatric Surgery

**DOI:** 10.1155/2019/3940739

**Published:** 2019-12-06

**Authors:** José Romeo Villarreal-Calderón, Ricardo X. Cuéllar, Martín R. Ramos-González, Nestor Rubio-Infante, Elena C. Castillo, Leticia Elizondo-Montemayor, Gerardo García-Rivas

**Affiliations:** ^1^Tecnologico de Monterrey, Escuela de Medicina y Ciencias de la Salud, Ave. Morones Prieto 3000, Monterrey, NL 64710, Mexico; ^2^Tecnologico de Monterrey, Centro de Investigación en Nutrición Clínica y Obesidad, Ave. Morones Prieto 300, Monterrey, NL 64710, Mexico; ^3^Tecnologico de Monterrey, Centro de Investigación Biomédica, Hospital Zambrano Hellion, TecSalud, San Pedro Garza Garcia NL 66278, Mexico

## Abstract

Low-grade chronic inflammation plays a pivotal role among other pathophysiological mechanisms involved in obesity. Innate and adaptive immune cells undergo systemic proinflammatory polarization that gives rise to an increased secretion of proinflammatory cytokines, which in turn leads to insulin resistance. Bariatric surgery is currently the most effective treatment for obesity, as it brings on significant weight loss, glucose metabolism improvement, and a decrease in systemic inflammation biomarkers. After bariatric surgery, several changes have been reported to occur in adaptive immunity, including reduction in CD4+ and CD8+ T cell counts, a decrease in the Th1/Th2 ratio, an increase in B regulatory cells, and reduction in proinflammatory cytokine secretion. Overall, there seems to be a major shift in several lymphocyte populations from a proinflammatory to an anti-inflammatory phenotype. Furthermore, increased antioxidant activity and reduced lipid and DNA oxidation products have been reported after bariatric surgery in circulating mononuclear cells. This paper highlights the shift in the adaptive immune system in response to weight loss and improved insulin sensitivity, as well as the interplay between immunological and metabolic adaptations as a result of bariatric surgery. Finally, based on data from research, we propose several mechanisms such as changes in adaptive immune cell phenotypes and their by-products, recruitment in adipose tissue, reduced oxidative stress, and modification in metabolic substrate availability as drivers to reduce low-grade chronic inflammation after bariatric surgery in severe obesity.

## 1. Introduction

Obesity is defined as an excess of body fat. Body mass index (BMI) has been the most widely used parameter to assess and classify the grade of obesity. The World Health Organization defines obesity as a BMI ≥ 30 kg/m^2^ [1]. In recent decades, obesity prevalence has risen to alarming levels. Global prevalence has increased from 3.2% in men and 6.4% in women in 1975 to 10.8% and 14.9%, respectively, in 2014 [2]. Obesity has been associated with metabolic disorders such as insulin resistance [3], dyslipidemia [4], and nonalcoholic fatty liver disease [5] and with endocrine conditions such as type 2 diabetes mellitus (T2DM) [6], polycystic ovarian syndrome [7], and vitamin D deficiency [8]. Despite a strong epidemiological association indicating an increased risk for obese subjects to suffer metabolic comorbidities, it should be noted that a proportion of the obese population has no manifest disorder (the so-called “metabolically healthy obese”), while also a relatively small but considerable proportion of normal-weight subjects may suffer from the metabolic conditions associated with obesity [9]. Obesity has also been related to autoimmune diseases such as rheumatoid arthritis [10], psoriasis [11], and systemic lupus erythematosus [12]. Furthermore, obesity increases mortality [13] and diminishes quality of life [14].

The treatment for obesity has proven to be a difficult challenge. Lifestyle changes including a change of diet and an increase in physical activity have been widely approved as the first-line options [15]. Several drugs, indicated as a complementary treatment to lifestyle changes, have proven to be effective in achieving a weight loss of 5% [16, 17]. However, weight regain is a common problem given that only about 50% of the subjects have been found to achieve a weight loss of at least 5% after 8 years of an intensive lifestyle intervention [18]. On the other hand, bariatric surgery has been regarded as the most effective long-term treatment for obesity [19]. According to the clinical guidelines from the American Society for Metabolic and Bariatric Surgery, surgical procedures for weight loss are indicated for patients with a BMI ≥ 40 kg/m^2^, a BMI ≥ 35 kg/m^2^ with at least one obesity-associated comorbidity, or a BMI ≥ 30 kg/m^2^ with either T2DM or metabolic syndrome [20].

Obesity is regarded as a low-grade inflammatory state characterized by an elevation of acute-phase reactants and proinflammatory cytokines. Inflammation is driven by the immune response, which is classified into innate and adaptive immunity. The innate response, mediated by neutrophils and macrophages that rapidly migrate to the inflamed tissues to try to eliminate the offensive agent, is nonspecific but fast-acting. On the other hand, the adaptive response is directed against a specific insult, mediated by T and B lymphocytes, which recognize specific epitopes with high affinity by the T cell receptor (TCR) or by antibody production, respectively. Both responses usually interact with and reinforce each other. For instance, macrophages act as antigen-presenting cells (APC) for T cells, and in turn, T cells secrete proinflammatory cytokines such as interferon-*γ*, which further activates macrophages [21, 22]. Specifically, the proinflammatory milieu associated with obesity is driven in part by adipose tissue macrophages, which are a component of the innate immune system. However, increasing recent evidence indicates that adaptive immune cells, such as T and B lymphocytes, play a crucial role in the activation and maintenance of such inflammatory state [23, 24]. The purpose of this review is to highlight the alterations in the adaptive immune system that follow bariatric surgery-induced weight loss and the potential underlying mechanisms associated with the inflammatory milieu and the insulin resistance status.

## 2. Low-Grade Chronic Inflammatory State in Obesity Is Driven by Both Innate and Adaptive Immune Cells

Obesity is associated with a low-grade chronic systemic inflammatory state characterized by elevation of acute-phase proteins such as C-reactive protein (CRP) produced by the liver [25] and IL-6, secreted by adipocytes and adipose tissue macrophages [26–28], as well as by an increase in TNF-*α* by adipose tissue [29]. Although early studies failed to find a significant increase in circulating TNF-*α* in patients with obesity [30], recent reports have observed higher serum TNF-*α* among obese subjects [31]. TNF-*α* has been shown to be mainly secreted by macrophages [32] that infiltrate adipose tissue creating crown-like structures (CLS) around necrotic adipocytes [33]. The role of adaptive immunity in obesity has been partly described. B lymphocytes have also been found within CLS in human adipose tissue, although their role is not completely understood [34]. In addition, adipose tissue from obese subjects has been found to contain an increased number of both CD4+ and CD8+ T lymphocytes, which also secrete proinflammatory cytokines such as TNF-*α* and IFN-*γ* [35]. Furthermore, increased waist circumference has been associated with increased expression of the activation markers CD25 and CD69 in T lymphocytes from adipose tissue [36]. Immune cells in peripheral blood have also been found to be related to inflammatory markers. Th1 cells are well known to secrete IFN-*γ*, a proinflammatory cytokine, while Th2 cells secrete IL-4, an interleukin with an anti-inflammatory role [23]. Peripheral blood mononuclear cells (PBMCs) have been shown to exhibit a proinflammatory secretory profile in obese subjects [37, 38]. Also, higher levels of the activation factor CD25 in T lymphocytes and increased Th1/Th2 ratios, correlated with insulin resistance assessed by the HOMA index, have been reported in obese subjects compared with lean or healthy overweight controls [39]. Furthermore, BMI has been found to be positively correlated with CD4+ effector memory T lymphocytes and negatively correlated with anti-inflammatory T regulatory (Treg) lymphocytes in severely obese subjects undergoing bariatric surgery [40].

Other specific T lymphocyte populations, such as mucosal-associated invariant T (MAIT) cells, have also been associated with the secretion of proinflammatory mediators. MAIT cells are innate-like T lymphocytes found in peripheral blood, intestinal mucosa, and the liver. Circulating MAIT cell numbers have been found to be reduced in patients with obesity and in those with T2DM. However, secretion of IL-17 by MAIT cells was increased in obese compared with the lean subjects. In addition, secretion of IL-2, granzyme B, and TNF-*α* was increased in T2DM. Noteworthy, both MAIT and CD8+ cells were found to be more abundant in omental adipose tissue than in peripheral blood in obese patients. These findings suggest a recruitment of MAIT cells by adipose tissue in obese subjects [41]. The polarization toward proinflammatory subpopulations of lymphocytes is a systemic phenomenon that has been observed in several tissues, including the skeletal muscle, liver, and pancreas [42].

The role of innate immunity in the development of obesity-associated low-grade chronic inflammation has been well studied. Mounting evidence indicates that despite the lack of an identified specific antigen, the adaptive immune system also participates in the development of this inflammatory state and exhibits proinflammatory polarization.

## 3. Inflammation Drives Insulin Resistance

Although a causative relationship between inflammation and insulin resistance is generally not regarded as proven, multiple evidence from studies in animal models and clinical trials that suggest that chronic inflammation may be a mechanism involved in the development of insulin resistance has been shown [43]. Adipose tissue-secreted TNF-*α* has been shown to induce insulin resistance in animal models [29] through activation of Janus N kinases (JNK) and serine phosphorylation of insulin receptor substrate (IRS) [44]. Both TNF-*α* and IL-6 have been associated with incident T2DM in case-control studies [45]. Furthermore, anti-TNF-*α* treatment has been able to reduce insulin resistance in rheumatoid arthritis [46] and in psoriasis [47]. Kinases have been shown to play a role in insulin resistance. IKK*ε* (I*κ*B kinase *ε*) and TBK1 (TANK-binding kinase 1) are noncanonical I*κ*B kinases that stimulate IFN-*α* and IFN-*β*. While these kinases have been shown to increase in response to a high-fat diet, their inactivation, either by genetic knockout or by pharmacological drugs, has been shown to prevent obesity and insulin resistance in murine models [48, 49]. Furthermore, a novel dual inhibitor of IKK*ε* and TBK1, amlexanox, has been shown to improve glycemic control in T2DM in a subset of patients with increased baseline inflammatory status [50]. In addition, IFN-*γ* has been shown to decrease insulin sensitivity in human adipose tissue by downregulation of PI3K (fosfatidilinositol-3-kinase), inhibiting the adipocyte secretion of the lipogenic enzymes, fatty acid synthase, and lipoprotein lipase, in response to insulin [35]. A more detailed description of the mechanisms by which inflammatory mediators lead to insulin resistance may be found in the revision by Chen et al. [51]. Besides inflammation, other mechanisms leading to insulin resistance in obesity include mitochondrial dysfunction, hyperinsulinemia through a negative feedback loop, lipotoxicity, endoplasmic reticulum stress, hypoxia, and oxidative stress [52]. Therefore, inflammation is a clinically relevant mechanism, among others, that contributes to insulin resistance associated with obesity and a potential source of pharmacological target for the treatment of insulin resistance and even T2DM in some patients.

## 4. Insulin Might Induce Changes in an Immune Cell Phenotype

Lymphocytes express both glucose transporter 1 (GLUT1) and GLUT3. Alterations in lymphocyte glucose uptake have been demonstrated in T2DM. Diet-treated subjects recently diagnosed with T2DM were shown to have increased peripheral lymphocyte glucose uptake at 15 minutes but have decreased uptake at 30 and 60 minutes compared with healthy controls. In contrast, insulin-treated patients with longer duration of disease were shown to have increased lymphocyte glucose uptake at all time points [53]. In lymphocytes from healthy subjects, *in vitro* treatment with insulin at 50 mIU/ml was shown to increase lymphocyte glucose uptake at 30 minutes, as well as GLUT3 and GLUT4 expression. These findings demonstrate that peripheral lymphocytes react to insulin stimulation and, therefore, could represent a model for the study of insulin resistance [54].

In addition, in an *in vitro* model, insulin has been described to be promoting anti-inflammatory Th2 differentiation in CD4+ lymphocytes, an effect likely mediated by ERK (extracellular-signal-regulated kinase) phosphorylation. Interestingly, while the insulin receptor was not shown to be detected in resting T cells, it was significantly upregulated upon activation in both CD4+ and CD8+ cells [55]. Furthermore, lymphocytes from obese subjects were not shown to increase pAKT (protein kinase B) intracellular levels or to decrease the Th1/Th2 ratio upon *in vitro* stimulation with supraphysiological concentrations of insulin, as the lymphocytes from lean controls did, indicating that lymphocytes from obese subjects have an impaired response to insulin [39].

These results indicate that lymphocytes respond to insulin stimulation, a fact not widely explored. Furthermore, in conditions characterized by insulin resistance, such as obesity and T2DM, lymphocytes have an impaired response to insulin. Finally, lymphocytes from obese subjects are resistant to insulin-mediated Th2 differentiation *in vitro*. Therefore, Th1 polarization seen in obesity may be partially caused by insulin resistance. Whether this is a relevant mechanism *in vivo* requires further study.

## 5. Bariatric Surgery Induces Significant Weight Loss

Lifestyle changes have been indicated as the first-line treatment option for people with obesity, but weight loss has proven to be rather small. In the Look AHEAD interventional study, a mean weight loss of 8.5% was achieved after one year of an intensive lifestyle intervention, but after an 8-year follow-up, only 50.3% were able to maintain a 5% weight loss [18]. In another lifestyle change interventional study, a 5% weight loss was shown to be sufficient in order to increase insulin sensitivity in the adipose tissue, liver, and skeletal muscle, but not to reduce systemic inflammation markers. A significant decrease in CRP was proven to decrease only after a progressive weight loss of more than 15%, although no significant changes were seen for IL-6, MCP-1, or white blood cell count [56]. In contrast, bariatric surgery has proven to induce a consistent significant and persistent weight loss. A pooled meta-analysis which included 25 studies concluded that bariatric surgery was more effective than nonsurgical interventions in achieving weight loss after a follow-up of one, two, and three years. Bariatric surgery was proven to achieve a weight loss of at least 20 kg higher compared with nonsurgical interventions [19]. A worldwide study estimated that bariatric surgery overall induces a weight loss of 30.5% at one-year follow-up [57].

**Figure 1:**
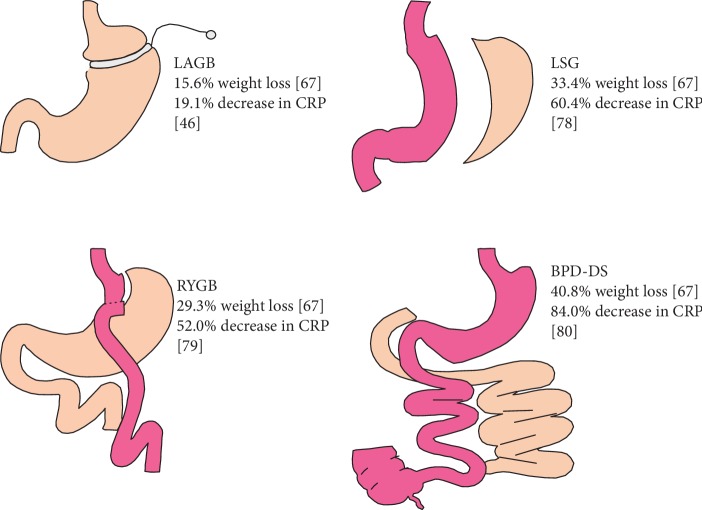
Graphic representation of bariatric surgical procedures to achieve weight loss and a % decrease in CRP. LAGB: laparoscopic adjustable gastric band; LSG: laparoscopic sleeve gastrectomy; RYGB: Roux-en-Y gastric bypass; BPD-DS: biliopancreatic diversion with duodenal switch; CRP: C-reactive protein. References in brackets. Further details in text.

Roux-en-Y gastric bypass (RYGB), laparoscopic sleeve gastrectomy (LSG), laparoscopic adjustable gastric banding (LAGB), and biliopancreatic diversion with duodenal switch (BPD-DS) are the most common bariatric surgical procedures (Figure 1). LAGB and LSG are traditionally regarded as merely restrictive methods, since the main effect of the operation is a reduction in gastric capacity. RYGB and BDP-DS induce a reduction in gastric capacity along with a malabsorptive component, given that a partial bypass of the small bowel is generated. The malabsorptive component is far more significant in BPD-DS due to the increased extent of the bypassed small bowel. Beyond their restrictive and malabsorptive mechanisms, LSG, RYGB, and BPD-DS lead to alterations in endocrine mechanisms by modulation of the secretion of gastrointestinal factors, such as ghrelin and glucagon-like peptide 1 (GLP-1), which have an effect on satiety and on glucose metabolism [58, 59].

While RYGB is widely regarded as the “gold standard” of bariatric procedures, BPD-DS is considered more effective, since it induces greater weight loss. However, its increased technical complexity and the higher morbidity risk limit its use. Regarding weight loss with these different procedures, LSG weight loss outcomes are similar to those of RYGB, while LAGB induces a more modest weight loss [58, 59]. In a retrospective study, a weight loss of 15.6%, 29.3%, 33.4%, and 40.8% was achieved after a 3-year follow-up using LAGB, RYGB, LSG, and BPD-DS, respectively [60] (Figure 1). Gastric imbrication or plicature, which involves folding and stitching the stomach into itself to adopt a tubular structure, is a relatively new surgical technique used for weight loss. Although there is little available data, short-term outcomes seem to be similar to those of sleeve gastrectomy [61]. In addition to weight loss, bariatric surgery is known to improve control of obesity-associated comorbidities, particularly T2DM and insulin resistance in the case of RYGB and BPD-DS, due to their endocrine component [59, 62].

## 6. Bariatric Surgery Is Associated with a Decrease in Systemic Inflammation Assessed by C-reactive Protein

Multiple studies have reported a decrease in CRP levels, a frequently assessed marker of systemic inflammation in the clinical setting after bariatric surgery. CRP has been shown to decrease from 1.15 to 0.34 mg/l at 6 months after either RYGB or LSG in a small cohort of Mexican subjects [63]. CRP was also found to fall from 0.68 to 0.55 mg/dl 3 months after LAGB, associated with a 13.9% weight loss, in 46 subjects [64]. In another study with a smaller sample size (*n* = 20), but a longer follow-up at 3 years, LAGB was shown to induce a weight loss of 30% and CRP was found to decrease from 7.6 to 3.4 mg/l [65]. CRP was found to be reduced from 5.3 to 2.1 U/ml one year after LSG in 30 morbidly obese women in which a 29.3% weight reduction was achieved [66]. Other authors have reported a decrease in CRP from 1.02 to 0.49 mg/dl in 20 obese subjects 3 months after RYGB. Noteworthy, no correlation was found between changes in CRP and changes in BMI. This lack of correlation points to a potential association of CRP with direct markers of fat mass or with caloric deprivation or malabsorption [67]. Finally, CRP was described to decrease from 11.9 mol/l to 4.9 mmol/l at 6 months, with further reduction to 1.9 mmol/l at 12-month follow-up in 70 subjects that underwent BPD-DS. Meanwhile, a 37.1% one-year weight loss was achieved [68]. Overall, all bariatric procedures were found to decrease CRP concentrations, although differences among the procedures might be attributable to the different follow-up periods and the varied baseline levels. It seems, however, that bariatric procedures that induce greater weight loss are also the most effective in reducing CRP levels and systemic inflammation (Table 1). However, other predictors of decreased CRP levels, such as adiposity markers or caloric deprivation, have been proposed.

## 7. Weight Loss Induced by Bariatric Surgery Is Associated with a Systemic Decrease in Oxidative Stress Final Products

Obesity has also been associated with high levels of oxidative stress in different studies. Obese women were found to have higher plasmatic concentrations of lipid peroxidation and protein carbonylation markers, compared with normal-weight controls. When measured 6 months after bariatric surgery, the levels of these markers were significantly reduced, although the concentrations did not reach those of the control group [69]. Short-term outcomes after biliopancreatic diversion have demonstrated an increase in glutathione-S-transferase and glutathione reductase activity in plasma, despite a decrease in glutathione peroxidase activity. Lipid peroxidation was found to increase initially 15 days after surgery, but afterwards, a significant decrease that remained below baseline levels 3 months after surgery was shown [70]. After a 1-year follow-up of bariatric surgery patients, a significant reduction in both lipid peroxidation and protein carbonylation, as well as an increase in nonprotein thiols and in whole blood antioxidant enzyme activity, was demonstrated [71]. Altogether, these results indicate an overall reduction in oxidative stress after weight loss induced by bariatric surgery.

Furthermore, bariatric surgery-mediated weight loss has been shown to reduce oxidative stress specifically in PBMCs, mainly composed of lymphocytes. Antioxidant enzyme activity has been reported to increase in PBMCs, while products of lipid and DNA oxidation have been shown to decrease after a one-year follow-up in morbid obese patients that underwent bariatric surgery [72, 73]. Previous work indicates that mitochondrial ROS are necessary for T cell activation [74]. ROS modulation has been reported to reduce cytokine secretion [75, 76]. Therefore, ROS reduction observed after bariatric surgery-induced weight loss should be expected to contribute to decreased proinflammatory cytokine secretion. However, its relevance in this specific clinical context has not been directly addressed.

## 8. Bariatric Surgery Drives a Shift in Adaptive Immune Cells Characterized by Changes in Lymphocyte Count, Phenotype, and Anti-Inflammatory Marker Secretion

Changes in lymphocyte count and variations in the phenotype, as well as in the secretion of their by-products, have been reported after bariatric surgery-induced weight loss. Shortly after gastric banding, a decrease in the Th1/Th2 ratio together with a reduction in Th1 and/or an increase in Th2 levels has been reported in T2DM and prediabetes, with improvement in glucose metabolism [77, 78]. B lymphocytes have also been shown to shift from an effector to a regulatory phenotype, suppressing proinflammatory cytokine secretion by T lymphocytes after RYGB [79]. In addition, the total number of CD4+ and CD8+ circulating T cells was found to be reduced after laparoscopic greater curvature plication in morbidly obese patients, suggesting a declined level of cell-mediated immune activity [80]. Likewise, a reduction in the count of B, T CD8+, and natural killer (NK) lymphocytes has been described after bariatric surgery in a subset of severely obese subjects with insulin resistance [81]. However, in a small sample of 20 severely obese women, no significant change in T CD4+, T CD8+, B, and NK lymphocyte subpopulations was found after bariatric surgery. When analyzed by the type of surgery, a change in CD4+ T lymphocytes was shown to correlate with changes in BMI in the RYGB subgroup, but not in the LAGB group, suggesting that more pronounced weight loss might be associated with a reduction in CD4+ T cells [82]. Using another procedure, four months after laparoscopic greater curvature plication, a decrease in CD4+ T cells from 38.2% to 29.3%, in CD8+ from 17.3% to 9.5%, and in leptin from 43.01 to 24.8 ng/ml was demonstrated in 20 subjects [80]. However, the association between the observed changes in lymphocyte populations and leptin was not evaluated in the study. Considering these results, a weight loss-associated decrease in the inflammatory phenotype lymphocyte differentiation is induced after the diverse bariatric surgery procedures.

Furthermore, Tfh cells, which play a crucial role in activating and differentiating B lymphocytes, have been shown to reduce the expression of activation markers and the secretion of proinflammatory cytokines after RYGB. Activation markers of Tfh cells were found to be downregulated 3 months after RYGB. The secretion of IFN-*γ*, IL-2, IL-4, and IL-17 by Tfh cells was also shown to decrease, while no significant change was observed in IL-10. Secretion of IL-10 was described to be higher after 72 hr incubation of Tfh post-RYGB plus staphylococcal enterotoxin B- (SEB-) pulsed autologous B cells compared with that of Tfh pre-RYGB plus SEB-pulsed B cells. In addition, Tfh IL-10+ was shown to promote the differentiation of naïve B cells toward an IL-10- and TGF-*β*-secreting phenotype, directly mediated by IL-10 itself. B cells isolated after RYGB were found to express more IL-10 and TGF-*β* [83]. These findings demonstrate an overall decrease in inflammatory cytokines and an overall increase in anti-inflammatory cytokines secreted by Tfh cells which may have a direct effect on the differentiation of anti-inflammatory B cells. Similarly, in another study, 3 months after the RYGB, B lymphocytes were found to shift from a proinflammatory IL-6+ phenotype to an anti-inflammatory IL-10+ phenotype, while T lymphocytes were shown to reduce the secretion of proinflammatory cytokines IL-17 and IFN-*γ*. Furthermore, coincubation of B and T cells showed that preoperative B cells stimulated proinflammatory cytokine secretion by T cells, while postoperative B cells inhibited this phenomenon [79]. These findings indicate that bariatric surgery induces changes in the lymphocyte phenotype from proinflammatory to anti-inflammatory that further impact other cell populations to regulate their inflammatory potential.

Lips et al. [84] reported outcomes in inflammatory status in obese and diabetic women 3 months after RYGB. Weight loss was shown to lead to a reduction in systemic CRP, total T cells, and helper T cells, but paradoxically, also to an increase in TNF-*α* systemic concentration. It is speculated that this later finding may reflect incomplete recovery from the surgical procedure itself and that longer follow-up periods are recommended to get a better picture of systemic inflammation after bariatric surgery [84]. In addition, decreased circulating MAIT cell numbers with surprisingly increased proinflammatory IL-17 secretion have been found in obese subjects compared with healthy controls. The lower numbers of MAIT cells in obese subjects have been attributed to increased activation and infiltration in adipose tissue. After bariatric surgery, the subjects were reported to present increased MAIT cell count in peripheral blood, but with no decrease in IL-17 secretion [41]. The relevance of the changes in MAIT cells and whether they reflect a reduction in their proinflammatory phenotype require further study.

## 9. Changes in Adaptive Cellular Immunity Elicited by Bariatric Surgery-Induced Weight Loss May Be Linked to Metabolic Improvement

**Figure 2:**
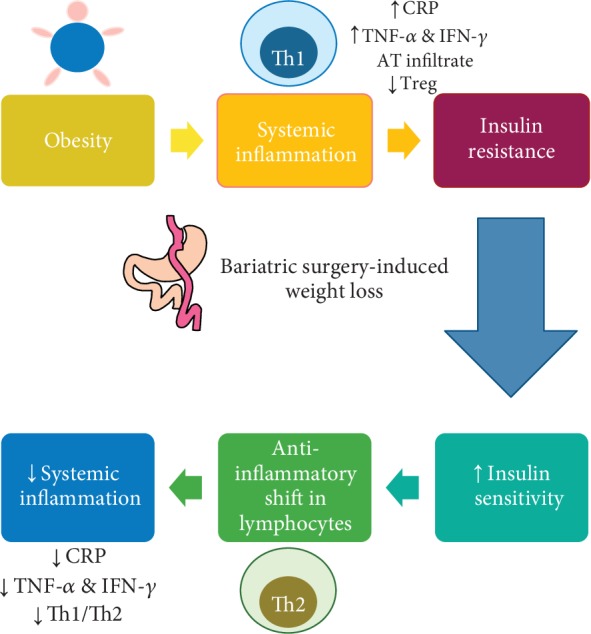
Potential mechanism of improvement in inflammatory status after bariatric surgery-induced weight loss. Obesity induces a systemic inflammatory status characterized by increases in C-reactive protein, proinflammatory cytokines such as TNF-*α* and IFN-*γ*, and an inflammatory infiltrate in adipose tissue (AT) and a decrease in circulating Treg lymphocytes. Inflammatory cytokines, among other mechanisms, induce insulin resistance in obesity. Bariatric surgery induces a significant weight loss that is associated with an increase in insulin sensitivity and a decrease in systemic inflammation. An important change in the lymphocyte phenotype is a decrease in the Th1/Th2 ratio after weight loss. It is possible that insulin effect on T cell differentiation may mediate inflammation resolution, at least partially.

The relationship of lymphocyte changes induced by bariatric surgery-induced weight loss with insulin sensitivity and metabolic alterations is crucial. However, available data is scarce and somewhat controversial, though pointing toward a positive link between them. While some studies have found changes in cell immunity to be related to glucose metabolism, others have not shown such a relationship. Viardot et al. evaluated changes in the immune system induced by weight loss after a 24-week-long restriction diet with laparoscopic gastric banding (LGB) performed at week 12 in obese subjects with either T2DM or impaired glucose tolerance (IGT) and in 10 healthy matched control subjects. The mean weight loss was found to be 5% at week 12 and 13.5% at week 24, while glucose control was described to improve significantly. Weight loss was shown to elicit a decrease in Th1 count and the Th1/Th2 ratio and to reduce the expression of CD69 and CD25 activation markers in lymphocytes. The decrease in CD69 and in the Th1/Th2 ratio was found to be associated with the reduction in BMI, but not with the HOMA-IR index or with fasting glucose [77]. However, other studies have shown a link between adaptive immune changes and metabolic parameters after bariatric surgery. The association between glucose metabolism and both systemic and adipose tissue inflammation was evaluated in 15 subjects with BMI above 35 kg/m^2^ and either T2DM or IGT who underwent LGB. Weight loss of 12.5% at week 12 was found to lead to lower fasting glucose levels and to a decrease in the Th1/Th2 ratio. A negative correlation between Th2 levels and fasting glucose concentration was also found [78]. Likewise, in another study, a negative correlation between Tfh IL10+ cell percentage change and BMI, glucose levels, and fat mass percentage was found after RYGB [83]. As inflammation is one of the mechanisms leading to insulin resistance and other obesity-related comorbidities, an increase in the anti-inflammatory lymphocyte subpopulation associated with decreased glucose levels after bariatric surgery might suggest that changes in cellular immunity after weight loss are linked to metabolic improvements. Noteworthy, the absolute numbers of B lymphocytes, CD8+ T lymphocytes, and NK lymphocytes have been reported to decrease after BPD-DS only in insulin-resistant subjects, indicating a potential relationship between these lymphocyte populations and insulin resistance. It is possible that some of these lymphocytes, particularly NK cells, contribute to the development of insulin resistance [81]. On the other hand, another unexplored possibility is that insulin resistance might be the driver responsible for the changes in lymphocyte populations, which would reverse after bariatric surgery-related improvement in insulin sensitivity (Figure 2). Insulin has an effect on lymphocyte differentiation, but the role played by insulin in bariatric surgery-induced weight loss, as well as the potential effects on other lymphocyte populations besides that on CD4+ T cells, requires further study. Changes in lymphocyte populations and cytokine secretion seen after bariatric surgery-induced weight loss are summarized in Table 2.

## 10. Shift to an Anti-Inflammatory Lymphocyte Phenotype after Bariatric Surgery and Weight Loss: From Insulin Resistance to Immunometabolism

As previously described in this paper, bariatric surgery has been well known to considerably reduce white adipose tissue mass, to improve insulin resistance and T2DM, and to induce a shift from the proinflammatory condition that characterizes obesity to an anti-inflammatory state. However, the mechanism by which weight loss induces activation of immune system cells is not completely understood. We propose three main mechanisms based on reports that describe the relationship between postoperative weight loss and changes in the immune system.

The first mechanism involves the association of adipose tissue and chronic inflammation. The role of adipose tissue in maintaining a low-grade chronic inflammation has been related to physiological stress in adipocytes as they progressively accumulate excessive fat. Increased release of inflammatory cytokines has been shown to elicit infiltration and activation of macrophages within the adipose tissue, which in turn alters the adipocyte secretion of adiponectin, leptin, and resistin. Thus, chronic insulin resistance is ensued and maintained [85]. Additionally, adipocyte death has been shown to induce immune cell activation and to initiate inflammation via macrophage activation [86]. The release of damage-associated molecular patterns (DAMPs) such as HMGB1 can lead to the activation of toll-like receptors (TLRs), and immune activation has also been described [87]. Also, differences in the localization of macrophages within adipose tissue between lean and obese fat mice have been found. In the lean group, interstitially spaced macrophages have been observed, while in the obese groups, macrophages are arranged in CLS [88], suggesting the presence of different subpopulations and paracrine activation. Therefore, the reduction of fat in the adipose tissue generated by bariatric surgery in the obese population would decrease the inflammatory stimulus driven by the interaction of immune cells and adipocytes.

**Figure 3:**
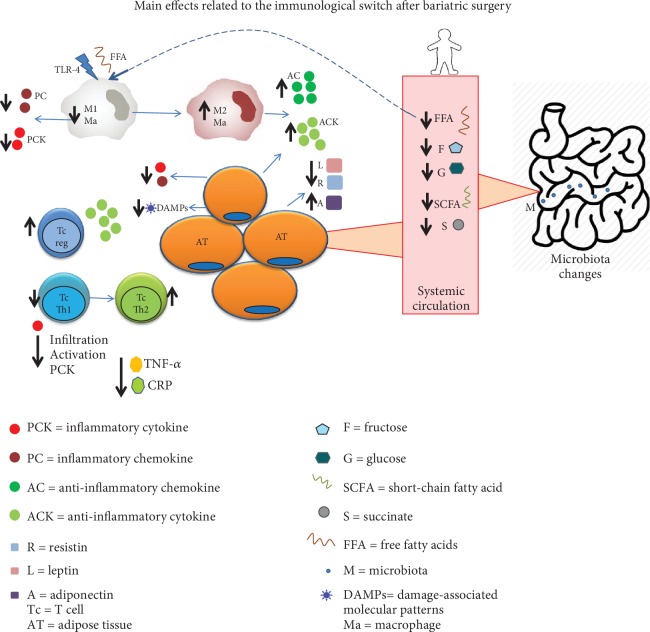
Main effects related to the immunological switch after bariatric surgery. Changes involved after weight loss include several molecular pathways: (i) a decrease in adipocyte proinflammatory mediators, (ii) changes in the activation and recruitment of immune cells in adipose tissue, (iii) modification in nutrient and metabolite absorption, and (iv) alterations in the intestinal microbiota.

The second mechanism involved in the relationship between postoperative weight loss and changes in the immune system is related to the activation and infiltration of immune cells. The reduction in the number and activation of infiltrating immune cells in the adipose tissue has been found to reduce the secretion of proinflammatory cytokines, such as CRP, IL-6, IL-1*β*, TNF-*α*, and IFN-*γ*, which in turn leads to a shift to an anti-inflammatory state and improves metabolic functions, contributing to weight loss. The interplay between the immune system and its associated effects on the metabolic status is a recent research field known as immunometabolism. Macrophages in lean adipose tissue have been described to have higher levels of arginase-1 and IL-10 expression, which are typical markers of anti-inflammatory M2 macrophages, compared with proinflammatory M1 macrophages found in obesity [89]. From this perspective, bariatric surgery could decrease infiltrating proinflammatory macrophages by reducing the differentiation of macrophages or by reestablishing the M1/M2 cell ratio, leading to a reduction in the infiltration of adaptive immune cells. Adaptive immunity in obesity has been shown to be affected by the infiltration of T and B cells into the adipose tissue, producing even more inflammatory cytokines, as demonstrated in obese mice [42, 90, 91]. After RYGB surgery, B cells have been shown to present a regulatory (IL-10) versus an effector (IL-6) profile and halt their previous role of sustaining T cell inflammation [79]. Bariatric surgery could modulate the increased activation of CD4+ and CD8+ T cells regularly observed in obese conditions and abate the level of both systemic inflammation and adipose tissue inflammation. In consequence, bariatric surgery can decrease or regularize the secretion of proinflammatory cytokines and insulin resistance, which in turn favors and improves glucose metabolism and adipogenesis (Figure 3).

The third mechanism involves the relationship of nutrients and immune cells. Metabolites such as glucose, free fatty acids, glutamine, and succinate are energy resources that can alter macrophage, neutrophil, and T cell functions [92]. Obesity is characterized by a continuous supply of these metabolites. The variations in these nutrient supplies observed after bariatric surgery could modulate the immune system and have been proposed as a key factor in immune differentiation. Bariatric surgery could modulate inflammation directly by decreasing the supply of nutrient metabolites through the inhibition of pathologic immune activation, hence leading to an anti-inflammatory state and improving metabolic functions. In particular, succinate has been shown to induce inflammation in macrophages *via* succinate receptor 1- (SUCNR1, also known as GPR91) mediated amplification of TLR signaling enhancing the secretion of IL-1*β* [93–94]. The metabolism of circulating fructose, which is mainly absorbed systemically by GLUT2 and GLUT5, has been associated with ATP depletion, oxidative stress, and inflammatory responses [95]. However, the cyclic guanosine-adenosine monophosphate (cGAMP) pathway has been described to be dysregulated, leading to the activation and induction of type I interferon synthesis induced by the STING receptor [87, 96]. Obesity is associated with an increase in T effector memory cells and a decrease in naïve T cells. This polarization in T CD4+ cells is driven by palmitate and is mediated by the PI3K-Akt pathway. The protein mTORc2 is involved in the activation of this pathway in an obese animal model [97].

**Figure 4:**
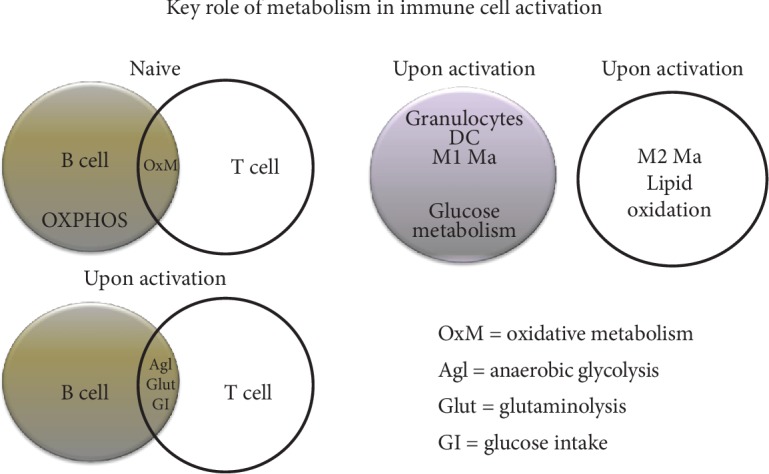
Metabolism modulates immune cell activation. Metabolic differences in *naïve* and activated T and B cells. Differences in energy supply between proinflammatory cells (M1 macrophages (M1 Ma)), dendritic cells (DC), granulocytes, and anti-inflammatory cells (M2 macrophages (M2 Ma)).

Short-chain fatty acids (SCFAs), by-products of microbial fermentation, can drive the differentiation of T cell subsets. The potential for butyrate and propionate produced by commensal bacteria in the gut to promote Treg cell differentiation has been previously demonstrated [98–100]. Fatty acid oxidation (FAO) has been described to enhance the functions of M2-polarized macrophages. Also, glucose metabolism has been implicated in granulocyte, dendritic cell, and M1-type macrophage activation. Th1 and Th2 immune responses have been suggested to be supported by the oxidation of fatty acids, inducing the development of the CD8+ memory phenotype, M2-like macrophages, and Tregs [101, 102]. Particularly, a rapid supply of ATP has been found to increase bioenergetics requirements in CD8+ T cells, improving their activity. Conversely, abundant lactic acid and glucose deficiency have been shown to impair the function of these cells. Treg cells have been described to depend on oxidative phosphorylation for energy source, as they rely on FAO and glutaminolysis for cell differentiation and proliferation, respectively [103–105] Figure 4. As a result of the weight loss induced by bariatric surgery, the nutrient supply deprivation mechanism is regulated through decreased activation of most immune cells, resulting in a decreased systemic inflammatory state.

Gut microbiota represents a potential link between bariatric surgery and SCFAs. Changes in gut microbiota have been observed in overweight and obese patients. Variations in the bacterial composition of the microbiota and their distribution along the bowel have been associated with metabolic alterations such as insulin resistance, low-grade inflammation, and adipocyte hypertrophy [106]. Reduced body weight and lower fat deposition have been found in a RYGB rat model compared with a sham group, attributed to a decrease in the absorption of SCFAs. SCFAs have been shown to be stored in the adipose tissue and to function as substrates for lipid and glucose metabolism, as well as immune cell modulators. As a consequence of fewer fatty acids being absorbed and stored in fat tissue, reduction in the secretion adipokines, growth factors, TNF-*α*, IL-6, leptin, and resistin in fat tissue ensues [107], as well as improvement in the adaptive immune system cells with reduction of both local and systemic inflammations. Transfer of gut microbiota from RYGB-treated mice to nonoperated germ-free animals has been reported to lead to weight loss and changes in specific SCFAs compared with microbiota transfer from sham to surgery animals. Thus, changes in SCFA concentration caused by modification in gut microbiota after bariatric surgery may alter metabolic functions in the host [108]. Diet and obesity have been shown to induce dysbiosis in microbiota favoring immunogenic bacterial products [109, 110]. Thus, immune system modulation after bariatric surgery might respond to changes in microbiota metabolic by-products.

## 11. Conclusion

Obesity is associated with a systemic low-grade inflammatory state in which cells from the innate and adaptive immune system increase proinflammatory cytokine secretion. Among other potential mechanisms, the inflammatory milieu leads to insulin resistance and metabolic comorbidities. An improvement in this deleterious condition is achieved through weight loss. Currently, bariatric surgery represents the most effective treatment option to induce significant and persistent weight loss. Weight loss induced by bariatric surgery leads to significant changes in adaptive immune cells. Both CD4+ and CD8+ T cell counts are reduced. Also, Tfh increases anti-inflammatory cytokine secretion, which leads to an increase in Breg cells. Anti-inflammatory cytokines, such as IL-10 and TGF-*β* produced by Breg cells, inhibit the secretion of the proinflammatory cytokines IFN-*γ* and IL-17 by T cells. Furthermore, a decrease in the Th1/Th2 ratio is also induced after bariatric surgery-mediated weight loss, probably related to improved insulin sensitivity. After bariatric surgery, immune cells develop stronger antioxidant capacity and reduce the level of lipid and DNA oxidation products. Oxidative stress is a known modulator of lymphocyte differentiation, metabolism, and proliferation, which improves after bariatric surgery. Changes in metabolic substrate availability after bariatric surgery, including glucose, succinate, and fatty acids, influence the adaptive immune response after bariatric surgery and still constitute a field that deserves further study. Palmitate, specifically, has been shown to promote T CD4+ effector memory cell differentiation in obesity. Mechanisms involved in bariatric surgery-induced changes in adaptive immunity include weight loss per se, caloric deprivation, substrate availability, insulin sensitivity, fatty acid, and metabolite concentration changes. A better underpinning of the specific influence of each of these mechanisms on immune cell modulation in the context of bariatric surgery-mediated weight loss in the patient with severe obesity represents an open area of study. Further research focused on the interplay between the immune system pathways and the metabolic processes after bariatric surgery may lead to developments for the treatment of obesity and its associated comorbidities.

## Figures and Tables

**Table 1:** Comparison of changes in systemic C-reactive protein observed after weight loss across different surgical procedures.

Population	Mean age (years)	Intervention	Follow-up (months)	Weight loss (%)	CRP reduction	Reference
36 subjects 88.9% F	37	RYGB/LSG	6	24.3	70.4%	[63]
46 subjects 87% F	40.6	LAGB	3	13.2	19.1%	[64]
20 subjects		LAGB	36	30.3	55.3%	[65]
30 subjects 100% F	40.3	LSG	12	29.3%	60.4%	[66]
70 subjects	41.3	BPD-DS	12	37.1%	84%	[68]

F: female; RYGB: Roux-en-Y gastric bypass; LSG: laparoscopic sleeve gastrectomy; LAGB: laparoscopic adjustable gastric band; BPD-DS: biliopancreatic diversion with duodenal switch; CRP: C-reactive protein.

**Table 2:** Summary of changes in lymphocyte populations and cytokine secretion observed after bariatric surgery-induced weight loss.

Population	Intervention	Main findings	Ref
13 subjects BMI ≥ 35 T2D or IGT Age: 35-65	12 weeks of DCR (860-1434 kcal/day) followed by LGB	↓ Th1/Th2 after DCR; changes maintained 12 weeks after LGB	[77]

15 subjects BMI ≥ 35 T2D or IGT Age: 35-65	LGB	↓ T cells and Th1/Th2, associated with lower FG, glucose AUC, and improved insulin secretion	[78]

9 subjects BMI: 35-38 Age: 45-61	RYGB	3 months after: B cells lose the capacity to support production of IL-17 and IFN-*γ* by T cells	[79]

20 subjects BMI: 37-45 Age: 25-50	Laparoscopic greater curvature plication	4 months after: ↓ CD4+ and CD8+ T cells and leptin	[80]

8 subjects BMI ≥ 40 With T2DM Mean age: 41.3	RYGB	3 months after: Tfh cells secreted: ↓ IFN-*γ*, IL-2, IL-4, and IL-17 and ↑ IL-10. Tfh IL-10+ promoted Breg cell differentiation and predicted better clinical response	[83]

69 subjects BMI ≥ 35 25% T2DM 30 subjects T2DM 67% obese	RYGB	↑ MAIT cells (potentially explained by ↓ peripheral infiltration); IL-17 remains	[41]

27 women 55.5% obese	RYGB	3 months after: ↓ IR, CRP, leptin, and T cells; ↑ TNF-*α*	[84]

58 subjects Nondiabetic BMI ≥ 40 63.8% IR Age: 18-60	6 weeks of VLCD followed by BPD	1 year after BPD: ↓ T cells and B cells in IR subjects Correlation: changes in CD8+ and HOMA index	[81]

20 women BMI: 36.4-68.2 Age: 25-90	LGB and RYGB	Correlation: changes from baseline in BMI and CD4+ cells only in the RYGB group at 3 months	[82]

DCR: dietary caloric restriction; BMI: body mass index; T2DM: type 2 diabetes mellitus; IR: insulin resistance; LGB: laparoscopic gastric banding; RYGB: Roux-en-Y gastric bypass; VLCD: very low-calorie diet; BDP: biliopancreatic diversion; ↓: decrease; ↑: increase; FG: fasting glucose; MAIT: mucosal-associated invariant T cells; IR: insulin resistance; CRP: C-reactive protein; Ref: reference.
